# Supplementary material for: WordCommentsAnalyzer: A windows software tool for qualitative research
Source: F1000Res. 2018 Sep 4;7:536. Originally published 2018 May 3. [Version 2] doi: 10.12688/f1000research.14819.2 (PMC6137411; doi:10.12688/f1000research.14819.2)
Supplement: Supplementary file 3 [file f1000research-7-17614-s0002.tgz › b2a79496-4ada-47c4-9682-a99ce867836d.zip!/Eight Iranian Health Journals/The Journal of Toloo-e-Behdasht_23.docx]

© The Journal of Toloo-e-Behdasht. This work is licensed under CC BY 4.0 (https://creativecommons.org/licenses/by/4.0/).

Volume & Issue: دوره 13 - شماره 4

Title: وضعیت بهداشت محیط مسکن مسلولین شهرستان یزد از نظر عوامل پیشگیری از انتقال بیماری های واگیر بر حسب استاندارد های سازمان جهانی بهداشت

http://tbj.ssu.ac.ir/article-1-1187-fa.html

مقدمه: یکی از عوامل مهم در ابتلاء شخص به بیماریها، وضعیت نامطلوب بهداشت مسکن می باشد. خانه تاریک، مرطوب و غیر بهداشتی زمینه را برای ابتلاء ساکنین آن به بیماری سل آماده می سازد. با توجه به نقش مسکن در برقراری شرایط برای انتقال بیماری سل، این مطالعه به منظور بررسی وضعیت بهداشت محیط مسکن مسلولین شهرستان یزد از نظر عوامل پیشگیری از انتقال بیماری های واگیر بر حسب استاندارد های سازمان جهانی بهداشت در سال 1389 طراحی شده است. روش بررسی: در این مطالعه توصیفی- مقطعی، اطلاعات مربوط به وضعیت بهداشت محیط مسکن کلیه بیماران مبتلا به سل شهرستان یزد به تعداد 86 مورد به طریق سرشماری و با تکمیل پرسشنامه به صورت مشاهده و اندازه گیری جمع آوری گردید. یافته ها: نتایج نشان داد که منازل مسلولین از نظر نوع ساختمان1/22 درصد خشتی و گلی، میانگین تراکم 55/3 نفردر اتاق، 50 درصد منازل مسکونی داری نورطبیعی نامناسب و 7/54 درصد دارای تهویه طبیعی نامناسب و 7/26 درصد دارای وسایل گرم کننده نامناسب بودند. همچنین 6/32 درصد کف اتاق، 6/32 درصد دیوار اتاق، 2/37 درصد سقف اتاق ها، 1/22 درصد دفع فاضلاب، 8/12 درصد آب شرب مصرفی، 9/20 درصد جمع آوری زباله، 6/11 درصد نگهداری حیوانات اهلی و 9/20 درصد نحوه دفع زباله از منزل غیر بهداشتی بودند. علاوه بر این منازل مسلولین 3/2 درصد فاقد توالت و 7 درصد فاقد حمام بودند. نتیجه گیری: با مقایسه ی وضعیت بهداشتی مسکن افراد مسلول با استاندارد های موجود، وضعیت این منازل نامطلوب ارزیابی گردید. و لذا اصلاح آن با اجرای برنامه های آموزشی جهت آگاه ساختن افراد از مزایای زندگی در مساکن بهداشتی در پیشگیری از ابتلا به بیماری سل را می طلبد. همچنین بایستی توجه دست اندر کاران تأمین مسکن و سازمان های مربوطه را برای کمک به بهبود مسکن افراد کم در آمد در جهت بهداشتی کردن آن ها جلب نمود.

Title: مقایسه آگاهی و نگرش دانش آموزان دبیرستانهای استان یزد در مورد ایدز در سال های 1383 و 1392

http://tbj.ssu.ac.ir/article-1-1188-fa.html

مقدمه: ایدز یک بیماری عفونی است که به عنوان چهارمین علت اصلی مرگ و میر در سطح بین المللی و مهلک ترین بیماری قرن حاضر شناخته شده است. امروزه دانش آموزان دبیرستانی بیشتر در معرض مواجهه با خطر ابتلا به HIV/AIDS هستند. هدف از این مطالعه مقایسه آگاهی و نگرش دا نش آموزان دبیرستانهای استان یزد در مورد ایدز در سال های 83 و 92 بوده است. روش بررسی: این پژوهش، مطالعه ای از نوع توصیفی و مقطعی می باشد که در آن 2146 دانش آموز از مقاطع مختلف تحصیلی در شهرستان های یزد (یزد، میبد، اردکان و مهریز) طی دو مرحله در سالهای تحصیلی 1383و 1392 مورد بررسی قرار گرفتند. داده ها توسط پرسشنامه جمع آوری شده و به وسیله نرم افزار آماری 16SPSS و آزمون های آماری t-test و ANOVA مورد تجزیه و تحلیل قرار گرفتند. یافته ها: داده ها نشان داد که آگاهی اکثریت دانش آموزان در سال 83 در حد خوب و در سال 92 در حد متوسط بوده است. نگرش اغلب دانش آموزان(3/36%) در سال 83 مثبت ولی در سال 92 نگرش اکثر دانش آموزان (1/70درصد) متوسط بوده است. میانگین درصد پاسخ های صحیح به راه های انتقال در سال 1383 برابر 36/84 بوده است، این میانگین در سال 1392 به 93/77 کاهش یافته است. داده های سال 92 نشان داد که بین نمره آگاهی و جنس، شهر محل سکونت و مقطع تحصیلی و بین نمره نگرش و جنس، سال تحصیلی، شهر محل سکونت و مقطع تحصیلی از لحاظ آماری ارتباط معنی داری وجود داشت(001/0P<). نتیجه گیری: داده های این مطالعه نشان داد که میزان آگاهی و نگرش دانش آموزان در سال 92 کمتر بوده و وضعیت نامطلوب تری نسبت به سال 83 داشته است. با توجه این که دانش آموزان مقاطع متوسطه گروه سنی در معرض خطر محصوب می شوند، لازم است برنامه های آموزش بهداشت به عنوان یک اولویت آموزشی در مدارس به منظور ارتقای سطح آگاهی و نگرش دانش آموزان مورد توجه قرار گیرد

Title: بررسی میزان کلیفرم کل، کلیفرم گرماپای و کلر باقی مانده آب استخرهای شنای سرپوشیده شهر کرمانشاه در سال های 90-1388

http://tbj.ssu.ac.ir/article-1-1189-fa.html

چکیده مقدمه:مطلوب بودن پارامترهای کلرباقیمانده، کلیفرم کل و باکتری های گرماپای نشان دهنده تامین حداقل استاندارد آب استخرها می باشد که در پایش کیفی آب آنها اهمیت فراوانی را دارد. لذا در این مطالعه، کیفیت آب 17 استخر سرپوشیده شهر کرمانشاه از نظر کلیفرم کل، کلیفرم مدفوعی و کلر باقیمانده مورد بررسی قرار گرفت. روش بررسی:این پژوهش یک نوع مطالعه توصیفی – تحلیلی می باشد. در این تحقیق، در کل 12 ماه سال به مدت سه سال پیاپی، کیفیت آب 17 استخر سرپوشیده شهر کرمانشاه با برداشت 618 نمونه از نظر کلیفرم کل، کلیفرم مدفوعی و کلر باقی مانده مورد بررسی قرار گرفت. کلیه شرایط نمونه برداری و انجام آزمایشها طبق روش استاندارد آزمایش های آب و فاضلاب انجام گرفت. یافته ها: نتایج نشان داد که مطلوبتر ین و نامطلوب ترین کیفیت آب از نظر کلر باقی مانده به ترتیب مربوط به استخرهای C‏ وL و (P و I) بود. مطلوبتر ین و نامطلوب ترین کیفیت نیز از نظر کلیفرم گرماپای مربوط به استخرهای (CوI) و (GوH ) می باشد. از نظر کلر باقی مانده، فروردین و اردیبهشت ماه کمترین مطلوبیت و مهر و آبان ماه بیشترین مطلوبیت و از نظر کلیفرم مدفوعی، مرداد و شهریور کمترین مطلوبیت و آذر و دی بیشترین مطلوبیت را به خود اختصاص دادند. روند مطلوبیت کیفیت آب استخرها از نظر کلر باقی مانده و کلیفرم مدفوعی از سال 1388 تا 1390 یک روند صعودی بوده و از نظر فصل، کمترین مطلوبیت مربوط به فصول بهار و تابستان و بیشترین مطلوبیت مربوط به پاییز و زمستان بود. از هر دو نظر کلرباقی مانده و کلیفرم مدفوعی، استخرهای صرفاً مردانه بیشتر مطلوبیت، نوع صرفاً زنانه ی آن کمترین مطلوبیت و نوع زنانه-مردانه، حالت بینابینی دو نوع فوق الذکر را دارا بود. نتیجه گیری: روند نظارتی و بازرسی مأمورین بهداشت محیطی در پایش کیفیت آب استخرها، تأثیر مثبتی در ارتقاء کیفیت آب استخرها در طی سال 1388 تا 1389 را نشان می دهد. با توجه به نتایج، هر چند که کیفیت آب استخرها از نظر پارامترهای مورد بررسی در حد تقریباً مطلوبی قرار دارد اما باید در فصول گرم بویژه در استخرهای صرفاً زنانه و استخرهای با منبع تأمین آب شهری نسبت به سایر استخرها، نظارت بیشتری صورت گیرد.

Title: بررسی کارآیی خاکستر زیتون تلخ در حذف کروم شش ظرفیتی از فاضلاب سنتزی صنعت آبکاری

http://tbj.ssu.ac.ir/article-1-1190-fa.html

مقدمه: کروم از جمله مهمترین فلزات سنگینی است که به صورت اکسیدهای سه و شش ظرفیتی در صنایعی مانند شیشه، سرامیک، دباغی ، چرم و بویژه در صنایع آبکاری و متالوژی کاربرد دارد. کروم شش ظرفیتی دارای مخاطرات بهداشتی اثبات شده ای مانند سرطانزایی است. هدف از این مطالعه بررسی کارآیی خاکستر زیتون تلخ(melia azedarach) در حذف کروم شش ظرفیتی از فاضلاب سنتزی صنعت آبکاری است. روش بررسی: این تحقیق یک مطالعه تجربی است که در آن اثر غلظت اولیه کروم، جرم جاذب و زمان واکنش بر حذف کروم شش ظرفیتی توسط جاذب بررسی شده است. جاذب مورد نظر از طریق سوزاندن میوه گیاه زیتون تلخ در شرایط آزمایشگاهی در دمای550 درجه سانتیگرادو زمان 2 ساعت تهیه و با استفاده از الکهای استاندارد ASTM با اندازه های مش بین 100-60 دانه بندی شد. مطالعه روی نمونه‌های سنتزی حاوی غلظت های 5و10 میلی گرم در لیتر کروم شش ظرفیتی انجام و غلظت مقادیر مجهول کروم شش ظرفیتی به روش اسپکتروفتومتری در طول موج nm 540 در حضور معرف دی فنیل کاربازاید تعیین شد. یافته ها: با توجه به نتایج، با افزایش جرم جاذب و زمان واکنش مقدار حذف کروم شش ظرفیتی افزایش و با افزایش غلظت اولیه کروم مقدار حذف کاهش یافته است. داده‌های این تحقیق نشان داد که جذب کروم شش ظرفیتی توسط خاکستر میوه زیتون تلخ در مدت زمان 180دقیقه به حالت تعادل رسیده و حداکثر میزان جذب حاصل می‌شود. نتیجه گیری:خاکستر زیتون تلخ جاذبی مؤثر در حذف کروم از فاضلاب سنتزی می باشد. به علاوه کاربرد این جاذب از نظر آماده سازی و هزینه بسیار ساده و ارزان بوده و کاربرد آن در مقایسه با بسیاری دیگر از جاذبهای طبیعی و مصنوعی دارای اولویت است.

Title: بررسی تاثیر میزان صدا و شدت روشنایی بر وضعیت بدن دانشجویان حین مطالعه در کتابخانه خوابگاههای پسرانه دانشگاه علوم پزشکی شیراز

http://tbj.ssu.ac.ir/article-1-1191-fa.html

چکیده مقدمه: صدا و روشنایی از جمله عوامل فیزیکی هستند که می توانند بر پوسچر افراد حین مطالعه تاثیرگذار باشند. با توجه به اهمیت عوامل فیزیکی یاد شده و تاثیر آنها بر وضعیت بدنی افراد، اندازه‎گیری و ارزیابی این دو پارامتر و تعیین ارتباط آنها با پوسچر افراد در سالن‎های مطالعه ضروری به نظر می‎رسد. این تحقیق با هدف اندازه‎گیری و ارزیابی صدا و روشنایی و تعیین ارتباط این دو عامل با پوسچر دانشجویان انجام شده است. روش بررسی: در این مطالعه مقطعی که از نوع توصیفی- تحلیلی می باشد، 167 نفر از دانشجویان مرد و مجرد خوابگاه‎های دانشگاه علوم پزشکی شیراز انتخاب و مورد مطالعه قرار گرفتند. اندازه‎گیری صدا و روشنایی بر اساس استانداردهای کمیته ملی بهداشت حرفه‎ای ایران انجام گرفت. همچنین ارزیابی پوسچر دانشجویان به روش RULA انجام شد. یافته ها: در جامعه مورد مطالعه، شدت روشنایی (83/114±88/433 لوکس) و تراز فشار صوت (646/4 ± 598/49 دسی بل) به دست آمد. در ارزیابی به روش RULA، 7/34% از افراد مورد مطالعه در سطح اولویت اقدامات اصلاحی 2، 3/41% در سطح 3 و 24% در سطح 4 قرار گرفتند. نتایج نشان داد که تراز فشار صوت با پوسچر دانشجویان دارای ارتباط معنی داری نمی‎باشد، اما بین شدت روشنایی و پوسچر دانشجویان ارتباط معنی داری مشاهده شد(001/0 p)، (701/0 - = R). نتیجه‎گیری: از میزان شدت روشنایی می توان به عنوان یکی از عوامل تاثیر گذار بر پوسچر دانشجویان هنگام مطالعه نام برد، بنابراین بهبود روشنایی می تواند به بهبود پوسچر دانشجویان و به دنبال آن افزایش راحتی آنها کمک شایان توجهی کند. خانه

Title: بررسی رابطه بین هوش هیجانی با رضایت زناشویی در متاهلین شهر یزد

http://tbj.ssu.ac.ir/article-1-1193-fa.html

مقدمه:امروزه هوش هیجانی در ابعاد زندگی افراد ،اهمیت بالایی داشته و مشخص شده 80 در صد موفقیت افراد در مراحل زندگی مربوط به هوش هیجانی و تنها 20 درصد مربوط به ضریب هوش آنها می باشد که در این بین یکی از مولفه های بسیار مهم رضایت زناشویی است که هوش هیجانی می تواند آنرا دستخوش تغییراتی نماید لذا تحقیق حاضر با هدف بررسی رابطه بین هوش هیجانی و رضایت زناشویی انجام گرفت روش بررسی: روش این پژوهش توصیفی تحلیلی (پیمایشی)می باشد.جامعه آماری شامل کلیه متأهلین شهر یزد به تعداد 101828 نفرمی باشند که براساس جدول مورگان وگریجسی388 نفر از طریق روش نمونه گیری خوشه ای تصادفی انتخاب گردیدند.جهت جمع آوری اطلاعات از دو نوع پرسشنامه هوش هیجانی شرینگ وپرسشنامه رضایت زناشویی اینریچ استفاده شد و تجزیه و تحلیل داده ها از طریق آمارهای توصیفی و استنباطی چون ضریب همبستگی پیرسون انجام گردید. یافته ها:در این بررسی از 388 نفر آزمودنی 5/63 در صدافراد زن و5/36 در صدمرد بودند.میزان تحصیلات اززیر دیپلم تا فوق لیسانس و بالاتر بودکه5/50 در صدافراد مدرک دیپلم وکمتر را داشتند. 5/49 در صدشغل آنهاآزاد و بیشترین گروه سنی( 6/43 در صد) بین سنین 35-25سال بودند.6/61 درصد آنهامدت ازدواجشان کمتر از 10 سال بود.براساس نتایج این پژوهش بین هوش هیجانی ورضایت زناشویی رابطه معنی داری وجود داردوبین مولفه های هوش هیجانی(همدلی،شناخت عواطف،خود کنترلی ومهارت اجتماعی)به تفکیک بارضایت زناشویی نیز رابطه معنادار وجود داشت(01/0=p) وبین متغیرهای دموگرافیک با هوش هیجانی و رضایت زناشویی در بعضی موارد رابطه معنی دار ی دیده شد. نتیجه گیری: با توجه به اینکه بین هوش هیجانی و رضایت زناشویی و بعضی از متغیرهای دیگر رابطه معنی داری وجود دارد بنابراین دارا بودن هوش هیجانی بر روند بهبود و تقویت کانون خانواده موثر بوده و به عنوان مولفه های مهم به حساب می آید. لذا باانجام آموزش و ارتقاء سطح هوش هیجانی در جوانان موجب بهسازی روابط بین فردی و در آینده رضایت زناشویی آنها خواهد گردید.

Title: بررسی میزان رضایتمندی گیرندگان خدمات از کلینیک تخصصی دندانپزشکی خاتم الانبیا یزد، سال1390

http://tbj.ssu.ac.ir/article-1-1194-fa.html

مقدمه: برای ارزیابی خدمات بهداشتی و درمانی، دستیابی و توجه به نقطه نظر بیماران ضروری است. هدف از مطالعه حاضر، بررسی رضایتمندی گیرندگان خدمت ازکلینیک تخصصی دندانپزشکی خاتم الانبیاء وابسته به دانشگاه علوم پزشکی یزد و برخی عوامل مرتبط با آن بود. روش بررسی: این مطالعه یک مطالعه توصیفی –مقطعی بود که بر روی 1380 نفر ازگیرندگان خدمات دندانپزشکی از کلینیک دندانپزشکی خاتم الانبیا که بصورت اتفاقی انتخاب شده بودند با روش مصاحبه تلفنی انجام شد. پرسشنامه شامل: سوالاتی درباره مشخصات فردی و برخی دیدگاه های بیماران در مورد دندانپزشکی، عوامل مربوط به کلینیک، درمان انجام شده، درمانگر و کادر درمانی بود. یافته ها با استفاده از نرم افزار SPSS مورد تحلیل قرار گرفت. یافته ها: میانگین و انحراف معیار نمره رضایتمندی بیماران در این مطالعه 2/0±3 از 4 بود. بالاترین رضایتمندی از نصب تابلوهای راهنما(6/99%) و رعایت بهداشت و کنترل عفونت(5/99%) و بیشترین نارضایتی از وضعیت پارکینگ(96%) و هزینه درمان (1/21%) عنوان شد.2/90% از افراد مورد بررسی اظهار نمودند که درصورت نیاز به درمان در آینده، مجدداً به این مرکز مراجعه خواهند نمود. نتیجه گیری: رضایتمندی مراجعین به کلینیک تقریباً کامل بود.نارضایتی در رابطه با پارکینگ و نیز بالا بودن تعرفه برخی درمانها از دیدگاه بعضی از بیماران، با توجه مسئولین مربوطه در دانشگاه و استان وفعالیت بیشتر سازمان های بیمه گر در زمینه خدمات دندانپزشکی قابل اصلاح می باشد.

Title: بررسی عوامل مرتبط با قصد استفاده از دوچرخه براساس تئوری رفتار برنامه ریزی شده در دانشجویان پسر دانشگاه علوم پزشکی یزد

http://tbj.ssu.ac.ir/article-1-1195-fa.html

مقدمه :استفاده ازدوچرخه به عنوان یک وسیله حمل و نقل درون شهری و ورزشی تا حدودی برای عموم امکان پذیر است. انجام فعا لیت های بدنی همیشه به عنوان یک راهکار مداخله ای حیاتی و مهم در پیشگیری و درمان چاقی وسایر بیماریهای متابولیسمی در سنین مختلف زندگی مدنظر می باشد. تئوری رفتار برنامه ریزی شده برای اینکه شامل باورها کنترلی رفتار است، برای مطالعه فعالیتهای ورزشی مناسب به نظر می رسد. در این مطالعه دیدگاههای دانشجویان به عنوان یک گروه در دسترس و تاثیر گذار بر هنجارههای جامعه در مورد استفاده ازدوچرخه مورد بررسی قرار گرفت. روش بررسی: این مطالعه مقطعی که به منظور بررسی دیدگاهها و عوامل مرتبط با قصد استفاده از دوچرخه توسط دانشجویان انجام شد، نظر 250 نفر از دانشجویان پسر ساکن خوابگاه های دانشگاه علوم پزشکی شهید صدوقی یزد درمورد استفاده از دوچرخه را براساس سازه های تئوری رفتار برنامه ریزی شده و به صورت مستقیم جویا شد و داده ها با استفاده از نرم افزار16 SPSS وآزمونهای آنالیز واریانس، همبستگی و رگرسیون تجزیه و تحلیل گردید. یافته ها: میانگین سنی شرکت کنندگان در مطالعه 83/2±23/0 بود و میانگین کل نمرات کسب شده در بخش های قصد رفتاری 9/3 ± 54/8، نگرش 97/2±2/15، هنجار ذهنی 9/2 ± 1/12ودر قسمت کنترل رفتاری درک شده 6/4 ± 5/1 گزارش گردید. در تحلیل رگرسیون انجام شده سازه های نگرش و هنجار ذهنی پیش بینی کننده معنی داری برای قصد رفتاری تشخیص داده شدند )766/28F= و001/0 P<، 297/0 (R2=. نتیجه گیری: بطور کلی با توجه به اینکه براساس نتایج مطالعه حاضر هنجارذهنی و نگرش بیشترین همبستگی و قدرت پیشگویی را با قصد استفاده از دوچرخه داشته اند به نظر می رسد برای ترویج رفتار دوچرخه سواری دربین دانشجویان، فعالیتهای فرهنگی به منظور ترویج فرهنگ دوچرخه سواری می تواند موثر واقع شود.

Title: بررسی همزمان شاخص های کارایی در بیمارستان های دانشگاهی و غیردانشگاهی با استفاده از مدل پابن لاسو در استان یزد طی سال 1391

http://tbj.ssu.ac.ir/article-1-1197-fa.html

مقدمه: شاخص های کارایی پرکاربردترین ابزار اندازه گیری عملکرد بیمارستان ها هستند، اما کاربرد این شاخص ها بصورت منفرد می تواند منجر به گمراه کردن اندازه گیری عملکرد بیمارستان ها شود. استفاده از شاخص های همزمان می تواند بسیاری از این مشکلات جهت اندازه گیری عملکرد بیمارستان ها را از بین ببرد. لذا این مطالعه با استفاده از مدل پابن لاسو به بررسی همزمان این شاخص ها در بیمارستان های دانشگاهی و غیر دانشگاهی استان یزد می پردازد. روش بررسی: مطالعه ی توصیفی حاضر بصورت مقطعی در سال 1391 در 20 بیمارستان استان یزد انجام شد. تعداد نمونه ها شامل 13 بیمارستان دانشگاهی و 7 بیمارستان غیر دانشگاهی بود. داده ها با مراجعه به معاونت درمان دانشگاه جمع آوری گردید و سه شاخص کارایی: درصد اشغال تخت، میزان گردش تخت و متوسط مدت اقامت محاسبه و بررسی همزمان آنها با استفاده از مدل پابن لاسو انجام شد. یافته ها: مدل پابن لاسو شامل چهار منطقه می باشد که از بین 12 بیمارستان دانشگاهی، 3 مورد (25%) در منطقه اول، 2مورد (16%) در منطقه دوم، 5مورد (33/3%) در منطقه سوم و 3 مورد (25%)در منطقه چهارم قرار گرفتند و از بین 7 بیمارستان غیر دانشگاهی، 3مورد (8/42%) در منطقه اول، 1مورد (3/14%) در منطقه دوم، 2مورد (6/28%) در منطقه سوم و 1 مورد (3/14%) در منطقه چهارم استقرار پیدا کردند. نتیجه گیری: بیمارستان های شهرستان ها نسبت به شهر یزد و بیمارستان های دانشگاهی نسبت به غیر دانشگاهی در منطقه سوم-کارا- تمرکز بیشتری دارند. از اینرو بازبینی در شیوه های موجود تخصیص منابع و ابداع راهکار هایی برای ارتقاء عملکرد بیمارستان های شهر یزد و غیر دانشگاهی ضروری می باشد.

Title: بررسی تاثیر آموزش بر اصلاح مشکلات رشد در دانش آموزان مدارس ابتدایی شهر پاوه

http://tbj.ssu.ac.ir/article-1-1198-fa.html

مقدمه: اختلالات رشد در دانش آموزان سنین دبستان از جمله مشکلات شابع دوران کودکی است که تاثیرات منفی ماندگاری بر وضعیت فیزیکی و هوشی کودک خواهند گذاشت. بر این مبنا مطالعه حاضر با هدف بررسی میزان انواع این اختلالات و انجام مداخله جهت کاهش آنها در دانش آموزان مقطع ابتدایی شهر پاوه انجام شد. روش بررسی: این مطالعه از نوع نیمه تجربی (مداخله ای آموزش محور)است که در طی 15 ماه در مدارس ابتدایی شهر پاوه و در دو مرحله به انجام رسید. ابزار مورد استفاده در این مطالعه، نمودارهای پایش رشد موجود در کشور بود که وضعیت قد و شاخص توده بدن به سن بر اساس آنها سنجیده می شد. برای تحلیل داده‌ها نرم‌افزار10 STATA استفاده گردید و در همه تحلیل‌ها مقدار برای P محاسبه و 05/0>P معنی دار در نظر گرفته شد. یافته ها: در این مطالعه، تعداد 574 دانش آموز در مطالعه شرکت نمودند که 5/51 درصد از آنان را دختر و 5/48 درصد از آنان را پسران تشکیل دادند. در بررسی های صورت گرفته در پیش آزمون مشخص گردید که 3/23 درصد از دانش آموان دارای مشکل لاغری،8/4 درصد دارای اختلال کوتاه قدی، 8 درصد دارای اضافه وزن و 6/10 درصد دچار اختلال چاقی می باشند. همچنین نتایج بدست آمده نشان داد که بطور کلی مداخلات صورت گرفته باعث ایجاد تغییرات مثبت گردیده اند و میزان آنها بخصوص در مورد لاغری و اضافه وزن به طرز معنی داری تغییر یافته است (001/.>P). همچنین مطالعه نشان داد که میزان تغییرات ایجاد شده در دختران بیشتر از پسران است (042/.=P). نتیجه گیری: مطالعه حاضر نشان داد که می توان با طراحی و اجرای مداخلات مناسب و جامع و پیگیری مداوم و هدفدار آنها، مشکلات و اختلالات رشد و تغذیه را بطرز مناسبی مرتفع نمود. بی تردید روشها و نتایج این مطالعه می تواند در عرصه های مشابه مورد استفاده قرار گرفته و مثمر ثمر واقع گردد.

Title: بررسی خواص ضد میکروبی نانوذرات اکسید روی سنتز شده به کمک امواج اولتراسونیک

http://tbj.ssu.ac.ir/article-1-1199-fa.html

مقدمه: معرفی یک عامل ضد باکتریایی قوی از اهمیت فراوانی در جهت کنترل باکتری‌های بیماری‌زا بویژه سویه‌های مقاوم در برابر آنتی بیوتیک‌ها برخوردار می باشد. هدف از این تحقیق سنتز نانوذرات اکسید روی به کمک امواج اولتراسونیک و بررسی خواص ضد باکتریایی آن بود. روش بررسی: ابتدا نانوذرات اکسید روی به کمک امواج اولتراسونیک سنتز شدند. ویژگی‌های ساختار فیزیکی و شیمیایی این نانوذرات مورد بررسی قرار گرفت. فعالیت ضدباکتریایی این نانوذرات بر روی دو سویه باکتری اشرشیاکلی و اﺳﺘﺎﻓﻴﻠﻮﮐﻮﮐﻮس اورئوس به کمک روش های استاندارد انتشار دیسک، چاهک گذاری، تعیین حداقل غلظت ممانعت کنندگی و تعیین حداقل غلظت کشندگی مورد بررسی قرار گرفت. یافته‌ها: بررسی فیزیکی و شیمیایی نانوذرات اکسید روی سنتز شده نشان داد که ذرات در ابعاد نانو، کروی شکل، بدون هیچ ناخالصی تولید شدند. حداقل غلظت بازدارنده رشد باکتری برای اشرشیاکلی برابر با mg/ml 125/0 و اﺳﺘﺎﻓﻴﻠﻮﮐﻮﮐﻮس اورئوس برابر با mg/ml 062/0 گزارش شد. حداقل غلظت کشندگی باکتری برای اشرشیاکلی برابر با mg/ml 500/0 و اﺳﺘﺎﻓﻴﻠﻮﮐﻮﮐﻮس اورئوس برابر با mg/ml 250/0 گزارش شد. در مقایسه دو روش چاهک گذاری و دیسک گذاری، قطر هاله عدم رشد در روش چاهک گذاری بیشتر از دیسک گذاری شد. قطر هاله عدم رشد در روش چاهک گذاری برای باکتری اشرشیاکلی527/1±66/18 میلی‌متر و اﺳﺘﺎﻓﻴﻠﻮﮐﻮﮐﻮس اورئوس 577/0±66/24میلی‌متر گزارش شد. نتیجه گیری: نانوذرات اکسید روی با درجه خلوص بالا به کمک امواج اولتراسونیک قابل سنتز بوده و خواص ضدباکتریایی قابل توجهی را در مقابل باکتری‌های اشرشیاکلی و اﺳﺘﺎﻓﻴﻠﻮﮐﻮﮐﻮس اورئوس دارند. باکتری اﺳﺘﺎﻓﻴﻠﻮﮐﻮﮐﻮس اورئوس در مقایسه با اشرشیاکلی حساسیت بیشتری را در مقابل نانوذرات اکسید روی از خود نشان داد.

Title: ارزیابی خطر سلامتی فلزات سنگین (کادمیوم، نیکل، سرب و روی) در سبزی جعفری برداشت شده از برخی مزارع شهر همدان

http://tbj.ssu.ac.ir/article-1-1200-fa.html

مقدمه: سبزیجات از اجزای مهم رژیم غذایی هستند و قرار گرفتن در معرض رژیم غذایی آلوده به فلزات سنگین از طریق مصرف سبزیجات، یک خطر برای سلامت انسان محسوب می‌شود. این تحقیق با هدف تعیین غلظت فلزات کادمیوم، نیکل، سرب و روی در خاک و جعفری و ارزیابی خطر سلامتی این فلزات در سبزی جعفری برداشت شده از برخی مزارع شهر همدان صورت گرفته است. روش بررسی: این تحقیق یک مطالعه کاربردی بوده، برای این منظور از جعفری و خاک 3 مزرعه در شهر همدان با 3 تکرار برای هریک از مزارع نمونه‌برداری به عمل آمد. در کل، در این بررسی تعداد 9 نمونه جعفری و 9 نمونه خاک مورد ارزیابی قرار گرفت. نمونه‌ها در آزمایشگاه با استفاده از روش هضم اسیدی، هضم شدند. سپس غلظت عناصر توسط دستگاه نشر اتمی قرائت گردید و برای تجزیه و تحلیل داده‌ها از نرم افزار SPSSورژن 19 با سطح معناداری 05/0 استفاده شد. یافته‌ها: میانگین غلظت کادمیوم، نیکل، سرب و روی در سبزی جعفری به ترتیب برابر14/1، 56/2، 65/16، 23/25 میلی‌گرم بر کیلوگرم و میانگین غلظت این فلزات به ترتیب در خاک برابر 23/0، 51/23، 85/20، 5/57 میلی‌گرم بر کیلوگرم است. یافته‌های این مطالعه نشان داد که میانگین غلظت فلزات کادمیوم و سرب در گیاه جعفری بالاتر از حد استاندارد (WHO/FAO)است. این در حالی است که میانگین غلظت فلزات نیکل و روی در گیاه جعفری کمتر از حد استاندارد (WHO/FAO) تشخیص داده شد. همچنین میانگین غلظت هر چهار فلز مورد بررسی در خاک مزارع کمتر از حد استاندارد (WHO/FAO) بوده است. از طرفی مقدار شاخص خطر و سلامت(HRI)برای فلز سرب در جعفری بیش از 1 بوده و برای فلزات کادمیوم، نیکل و روی کمتر از 1 می باشد. نتیجه‌گیری: با توجه به یافته‌های مطالعه، جعفری کشت شده در این منطقه به فلزات سنگین کادمیوم و سرب آلوده بوده‌است و نتایج، حاکی از انتقال بسیار ساده این فلزات از خاک به جعفری است که برای مصرف روزانه انسان سالم نبوده و شاخص خطر و سلامت (HRI) بیش از 1 برای فلز سرب، نشان دهنده خطر بالقوه بهداشتی این فلز در ارتباط با مصرف جعفری آلوده در رژیم غذایی روزانه انسان است.

Title: بررسی کمی و کیفی پسماندهای تولیدی بیمارستان بعثت شهر سنندج در سال 1390

http://tbj.ssu.ac.ir/article-1-1201-fa.html

مقدمه: مدیریت صحیح زایدات بیمارستانی به لحاظ ایجاد آلودگی های زیست محیطی و بروز عفونتهای بیمارستانی نقش بسزایی در ارتقا بهداشت جامعه دارد. یکی از اساسی ترین طراحی و اجرای یک برنامه صحیح مدیریت مواد زاید بیمارستانی آگاهی از مقادیر کمی و کیفی پسماندهای بیمارستانی می باشد. مطالعه حاضر با هدف تعیین خصوصیات کمی و کیفی پسماندهای تولیدی در بیمارستان بعثت شهر سنندج انجام پذیرفت. روش بررسی: در این مطالعه که از نوع توصیفی- مقطعی می‌باشد در بیمارستان بعثت شهر سنندج در سال 1390 انجام شد. برای تعیین مقدار کمی و کیفی پسماند های تولیدی آنالیز انواع پسماندها هر ماه در یک نوبت و به مدت یک سال صورت پذیرفت. یافته ها با کمک شاخص های توصیفی ارائه شد. یافته ها: میانگین کمیت کل پسماند تولیدی در بیمارستان مورد مطالعه 5/1191 کیلوگرم در روز بود. که این مقدار برای پسماندهای عمومی (شبه خانگی) و پسماند های عفونی وخطرناک به ترتیب 65 (54/54%)،750/541 (46/45%) کیلوگرم در روز بود. با توجه به وجود 370 عدد تخت فعال در این مرکز، سرانه تولید زباله به ازای هر تخت فعال در روز 22/3 کیلوگرم برآورد شده است. بیشترین میزان زایدات عفونی و خطر ناک مربوط به بخش ICU2با72 /0 کیلوگرم در روز به ازای هر تخت فعال می باشد. آنالیز کیفی کل پسماند های بیمارستانی تولیدی نیز نشان داد که پلاستیک با 46 % بیشترین درصد وزنی را دارد. میانگین وزن مخصوص کل پسماندهای تولیدی بیمارستانی برابر با 75/45 کیلوگرم بر مترمکعب بدست آمد. نتیجه گیری: با توجه به درصد بالای پسماندهای عفونی و خطر ناک، می توان نتیجه گرفت که علی رغم تلاش فراوان در خصوص تفکیک پسماندهای بیمارستانی، هنوز مشکلاتی در این رابطه وجود دارد که نیازمند توجه بیشتر مسئولین و نیز استفاده از روش های نوین، جهت بی خطرسازی پسماند ها قبل از خروج از بیمارستان می‌باشد.

Title: بررسی کارایی کربن فعال دانه ای در کاهش COD ناشی از آلودگی آب با گازوییل

http://tbj.ssu.ac.ir/article-1-1202-fa.html

چکیده مقدمه: آلودگی با مواد ‌نفتی اثرات شدیدی روی منابع آب‌آشامیدنی، محیط‌زیست، خاک و در نهایت سلامت انسان‌ها دارد. از اهداف مهم مهندسین بهداشت محیط حذف و خارج کردن آلاینده‌ها از محیط می‌باشد. یکی از روش‌های موثر در حذف آلاینده‌ها فرآیند جذب سطحی است. در این مطالعه کارایی فرآیند جذب ‌سطحی با کربن فعال‌ دانه‌ای جهت کاهش اکسیژن مورد ‌نیاز شیمیایی ناشی از مقادیر کم گازوییل در آب ‌ بررسی شده است. روش ‌بررسی: این تحقیق، یک مطالعه تجربی است که در مقیاس آزمایشگاهی انجام شد. فرایند جذب به روش پیوسته در حجم 100 میلی لیتر در ارلن مایر درب دار انجام و متغیرهای موثر چون غلظت آلاینده (1 و 2 درصد حجمی) و زمان تماس (60، 30، 20، 10، 5، 2 دقیقه)، بهینه شدند. در تمام آزمایش‌ها pH نمونه‌ها (85/7) و مقدار جاذب 1 گرم در 100 میلی‌لیتر، ثابت بود. مقدار COD با روش رفلکس برگشتی مطابق با روش D 5220 استاندارد آزمایش‌های آب و فاضلاب اندازه گیری شد. برای تعیین ایزوترم جذب داده‌های به‌دست آمده را با سه مدل فروندلیچ، لانگمیر و تمکین برازش شدند و نمودارها با نرم‌افزارExcel رسم شدند. یافته‌ها: نتایج این مطالعه نشان داد متوسط درصد حذف گازوئیل در غلظت یک و دو درصد به‌ترتیب 17/3± 64/93 و 76/22± 7/53 بود که این تفاوت از نظر آماری معنادار است ( 05/0P<). همچنین ظرفیت جذب کربن در غلظت یک درصد و دو درصد به‌ترتیب 71/53 ± 28/264 و 17/87 ± 85/199 میلی گرم بر گرم به دست آمد که این تفاوت از نظر آماری معنادار نیست (05/0P >). مدل فروندلیچ بهترین ایزوترم برای فرایند حذف گازوئیل با کربن‌فعال (84/0R2=) به دست آمد و سینتیک واکنش از سینتیک شبه‌درجه دوم پیروی کرد. نتیجه‌گیری: نتایج نشان داد که کربن‌فعال جاذب مناسبی جهت کاهش COD ناشی از آلودگی مقادیر کم گازوییل در آب است. استفاده از این جاذب می‌تواند در زمان 30 دقیقه اولیه به خوبی COD ناشی از آلودگی آب با گازوئیل را کاهش دهد.

Title: ارزیابی جو ایمنی در یکی از معادن استان یزد

http://tbj.ssu.ac.ir/article-1-1203-fa.html

چکیده مقدمه: امروزه با توسعه تکنو لوژی حضور و نقش نیروی انسانی در محیط‌های صنعتی پررنگ‌تر شده است. از این رو اهمیت داشتن فرهنگ ایمنی روز به روز بیشتر می‌شود. جو ایمنی به عنوان زیر مجموعه‌ای از فرهنگ ایمنی به بررسی ادراکات و استنباط‌های کارکنان در مورد محیط کار، سطح علاقه مدیریت به ایمنی و اقدامات مربوط به ایمنی و میزان مشارکت در کنترل ریسک می‌پردازد. و از طرفی کار در معدن جزء خطرناک‌ترین مشاغل شناخته شده است. لذا این مطالعه با هدف ارزیابی جو ایمنی در یکی از معادن استان یزد در سال 1391 انجام گرفت. روش بررسی: پژوهش حاضر، مطالعه‌ای توصیفی از نوع مقطعی بود. جامعه مورد بررسی تمامی کارکنان شاغل در بخش عملیات یکی از معادن استان یزد(تعداد32 نفر) تشکیل می‌دادند. ابزار گردآوری اطلاعات در دو بخش پرسشنامه استاندارد بیست سوالی جو ایمنی با روایی و پایایی معتبر (77/0α=) در هفت حیطه شامل تعهد مدیریتی، ارتباطات درباره مسائل ایمنی، محیط ایمن، مسئولیت پذیری مدیران، درک خطر ، رضایت شغلی و آگاهی و هشیاری نسبت به موضوعات ایمنی که پاسخ‌ها به صورت طیف لیکرت پنج درجه‌ای به صورت کاملا مخالفم (1 نمره) تا کاملا موافقم (5 نمره) بود. بخش دوم سوالات جمعیت شناختی و زمینه‌ای شامل: سن، وضعیت تاهل، سابقه کار، میزان درآمد ماهیانه، تحصیلات، مدت زمان کار و شیفت کاری بود. داده‌های جمع آوری شده به صورت مصاحبه توسط مسئول ایمنی و محیط زیست معدن به وسیله نرم افزار آماری SPSS ویرایش هجدهم و با کمک آزمون‌های توصیفی و تحلیلی چون ضریب همبستگی پیرسون، آنالیز واریانس یک طرفه، تی تست مستقل و آنالیز رگرسیون خطی در سطح معناداری 05/0 مورد آزمون قرار گرفتند. یافته‌ها: در این مطالعه1/28% مجرد، 9/71% متاهل و 6/40% دارای سطح تحصیلات ابتدایی و راهنمایی، 9/21% دیپلم و 1/28% لیسانس و بالاتر بودند. میانگین نمره هر یک از حیطه‌های تعهد مدیریتی66/2±09/11، ارتباطات 36/2±50/7، محیط ایمن 92/1±09/8، مسئولیت پذیری مدیران 58/1±56/6، درک خطر 56/1±43/8، رضایت شغلی 58/1±59/4 و آگاهی و هشیاری نسبت به موضوعات ایمنی 27/1±25/4 بود. میان میانگین سن و حیطه‌های تعهد مدیریتی (008/0=p ،458/0- =r)و ارتباطات درباره مسایل ایمنی (027/0=p ،90/0-r= ) همبستگی معناداری وجود داشت. حیطه‌های درک خطر و آگاهی و هشیاری نسبت به موضوعات ایمنی پیشگویی کننده رضایت شغلی کارگران بودند. نتیجه گیری: نتایج حاصل از این مطالعه نشان داد وضعیت حیطه‌های مختلف جو ایمنی از نظر کارکنان معدن مورد بررسی در سطح متوسطی قرار دارد.و این نشاندهنده فضای ایمنی متوسط در معدن مورد بررسی می باشد که می بایست توسط مدیران و مسئولان مربوطه مورد توجه قرار گیرد
